# Visual communication of public health data: a scoping review

**DOI:** 10.3389/fdgth.2025.1555231

**Published:** 2025-04-24

**Authors:** Michael Arthur Ofori, Stella Lartey, Polina Durneva, Niharika Jha, Nidhi Mittal, Shongkour Roy, Zebunnesa Zeba, Stella Chirwa, Nichole Saulsberry-Scarboro, Michelle Taylor, Ashish Joshi

**Affiliations:** ^1^ School of Public Health, University of Memphis, Memphis, TN, United States; ^2^ Department of Public Health Sciences, College of Behavioral, Social and Health Sciences, Clemson University, Clemson, SC, United States; ^3^ Department of Management Information Systems, Fogelman College of Business & Economics, University of Memphis, Memphis, TN, United States; ^4^ Shelby County Health Department, Memphis, TN, United States

**Keywords:** visual communication, interactive dashboards, data visualization, health data, health communication

## Abstract

**Introduction:**

Visual communications (VC) play a crucial role in effectively conveying public health data to diverse audiences, including policymakers, healthcare professionals, and the general public. Although the U.S. government invests heavily in health data and data accessibility, health data are not entirely accessible or easily understood. This can be attributed to data sharing and visualization challenges. VC challenges have created public health information gaps which are compounded in emergencies such as the COVID-19 pandemic, potentially impacting poor health outcomes and increasing health inequities.

**Objective:**

To examine visualization tools and techniques effective for public health visual data communication.

**Methods:**

A scoping review was conducted to summarize the available evidence related to visualization techniques and tools for public health visual data communication as well as related principles and best practices. Original peer-reviewed articles published in English that involve visualization, user-centered design of visual public health applications/interfaces, visual analytics, infographics, or dashboards from PubMed database from 2020 to 2024 were included. Also, review articles, commentaries, editorials, posters, systematic and scoping articles were excluded from this review. In all, twenty-eight (28) studies were included.

**Results:**

There were 25 different visualization techniques identified which included charts and graphs (e.g., bar charts, line charts, pie charts, bubble charts, box plots, scatter plots), maps (e.g., choropleth maps, hotspot maps, and heatmaps), and specialized visualizations (e.g., sunburst diagrams, alluvial plots, upset plots, circos). These visuals were displayed employing different programming and statistical tools and libraries like R, Python, Power BI, Tableau, ArcGIS, and custom web-based applications. The visuals measured different types of data accessibility, pattern and trends identification, association and relationships of univariate and bivariate data, as well as exploring multidimensional forms of health data. The visualizations were applied in different public health domains, such as HIV prevention and care, public health communication, interventions, surveillance, policy measures and decision-making, and improving health education.

**Conclusion:**

Dashboards and web-based tools combined with static visualizations like charts, maps, or specialized plots can help with data exploration, pattern recognition, and dissemination of health information. Effective communication of public health data promotes informed decision-making, creates awareness, and leads to improved and better health outcomes.

## 1 Introduction

Visual communication (VC) is the skill of arranging and presenting complicated data in clear and understandable graphic pieces. According to Cantor (1), visual communication involves representing data and information in various forms, such as graphs, charts, diagrams, and pictures. VC plays a crucial role in effectively conveying public health data to diverse audiences, including policymakers, healthcare professionals, and the general public (2). VC of health data using maps, graphs, and charts dates to the late 18th century. The very earliest ones include the John Snow's 1,854 map of the outbreak of cholera in London. The work and map of John Snow persuaded the local council to disable the water pump and led to changes in public health policies. Furthermore, the Rose diagram developed by Florence Nightingale in 1,858 is worth mentioning. Nightingale used a polar-area diagram, also known as a coxcomb chart, to illustrate the relationship between sanitary conditions and soldiers' deaths compared to deaths from battlefield wounds. Nightingale showed that visual communication of data can positively impact health policies and persuade governments.

Effective visual communication can help simplify complex information, highlight patterns and trends, and ultimately enhance decision-making processes related to public health issues (3). The health sector has seen phenomenal growth in data gathered from millions of people, each uniquely identifiable or anonymized and pooled together. According to RBC Capital Markets, approximately 30% of the world's data volume is generated by the healthcare industry. By 2025, the compound annual growth rate of data for healthcare will reach 36% (4). The global market of healthcare data analytics was estimated to grow 3.5 times in just six years, from $11.5 billion in 2019 to $40.8 billion by 2025 (5).

The U.S. government invests a significant amount of budget in health and data accessibility, and it is estimated to reach $19.9 billion by the end of 2024. Despite the effort, health data are not entirely accessible and understood due to data sharing and visualization challenges (3). Visual communication challenges such as inadequate visual designs, literacy level, limited resources, cultural and contextual differences, misinformation, and disinformation have created public health information gaps which are compounded in emergencies such as the COVID-19 pandemic. These challenges also potentially impact poor health outcomes and increase health inequities (6).

Static data visualizations have been employed in the field of public health for a long period, dating back to when John Snow utilized a map to investigate the source of a cholera outbreak in the 1850s, and continuing through to the present day (7). Interactive visualization tools are external resources fundamentally designed to aid and augment users' processes of exploring and deriving meaning from visually represented data (8). While the use of visualizations to depict and convey health data has seen a rise, a systematic review carried out by Carroll et al. revealed that static graphics continue to be the predominant approach for representing health data (9).

A variety of visualization techniques like charts, graphs, infographics, geographic maps, and dashboards have been employed to communicate different types of health data. According to (10), graphing vital signs data can rapidly detect changes in physiological measurements, indicating the need for intervention and demonstrating the effectiveness of treatments. Recently, visualizations have taken various forms, scales, colors, and dataset sizes. VC includes static visuals such as charts (bar, line, scatter plots), infographics, diagrams, and maps (11); interactive visuals like dashboards, data exploration tools, and dynamic visualizations (12); and storytelling visuals like visual narratives, animations, and video presentations (13).

In healthcare, VC remains a powerful tool helping professionals easily comprehend and interpret large data volumes, enabling informed clinical decisions, better patient outcomes, and efficient resource planning. To create effective healthcare data visualizations, it's crucial to select the appropriate visualization type, design for the target audience, using clear labeling, highlight key insights, and incorporate interactive and customizable features (10). Effective communication of public health data promotes informed decision-making, creates awareness, and leads to improved and better health outcomes. As health-related datasets become more available and complex, there is a more pressing need for appropriate visualization techniques and tools. As a result, this review presents a comprehensive overview of the current state of visual communication in public health. The review focuses on examining the diversity of visual representation methods used in public health data and analyzing the types of visualizations (e.g., interactive dashboards, statistical charts, geographical maps). It also identifies and list the programming languages and development tools used to create public health visualizations. Lastly, it assesses the measures and applications of these visuals in public health.

## 2 Methodology

### 2.1 Searched strategy

We conducted a review of peer-reviewed published articles in the month of February (starting from the 7th) 2024. We intentionally focused on peer-reviewed articles to ensure scientific rigor and systematic validation of visualization techniques as an initial exploration of the topic. The approach used in this study was compliant with the original Arksey and O'Malley Framework. These techniques involve formulating research questions, identifying appropriate research papers, selecting the appropriate study, organizing, and charting data, and compiling, presenting, and summarizing the results (14) which aligns with the current study. The Arksey and O'Malley Framework provides a systematic and rigorous methodology for conducting scoping reviews. It offers a clear, step-by-step process that helps develop a comprehensive and transparent approach to mapping existing literature. The framework's ability to identify the research question, relevant studies, study selection, charting the data and collating, summarizing, and reporting the results makes it an ideal choice for this work.

**Figure 1:**
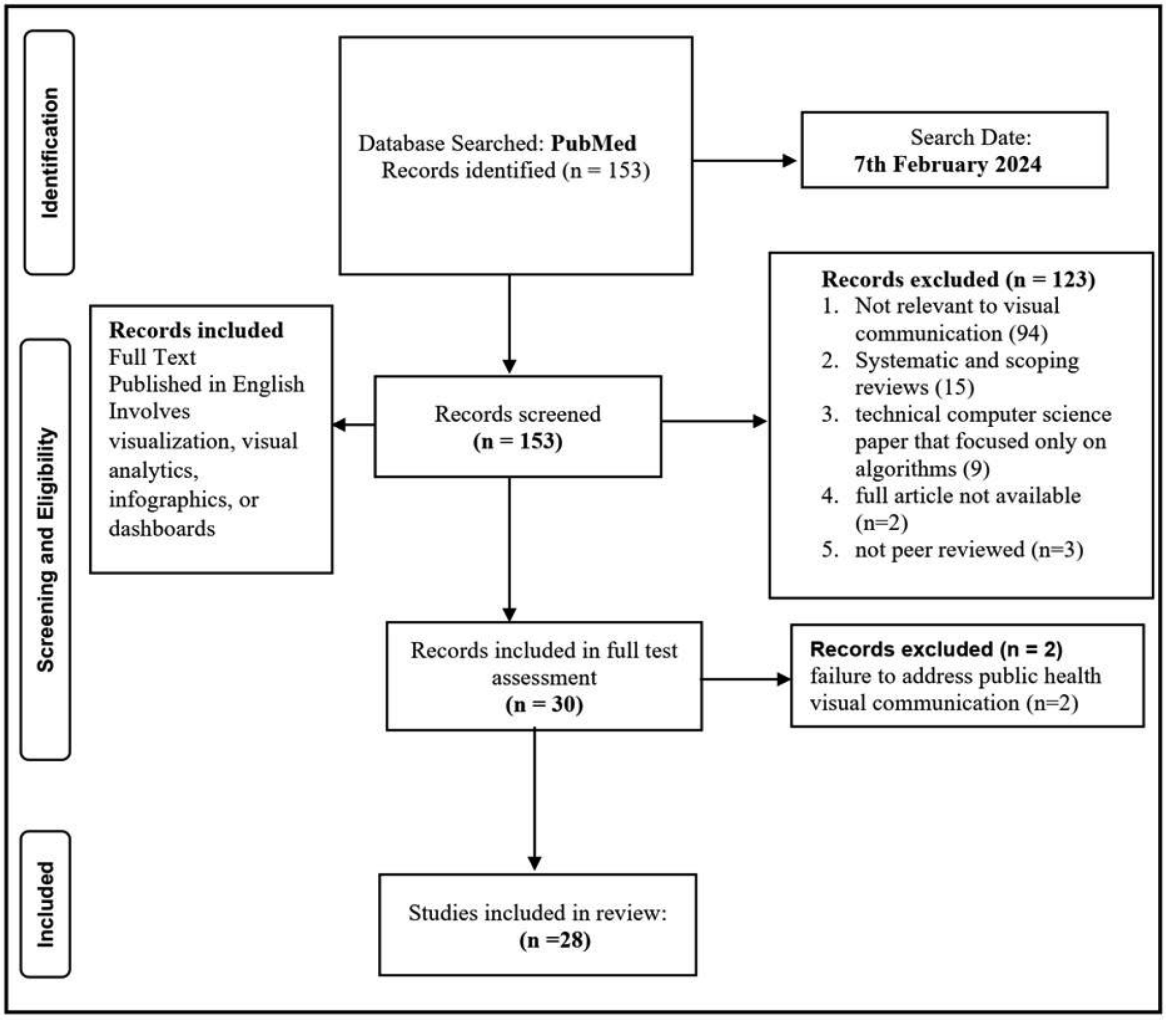
PRISMA diagram showing the flow of the study selection and search approach.

The study reported details of the databases searched, the search strategy used, the eligibility criteria, and the process of study selection. The review used the PRISMA-ScR (Preferred Reporting Items for Systematic Reviews and Meta-Analysis extension for Scoping Review) checklist to guide the reporting of the literature search (as shown in Figure 1). This approach is thought to be appropriate for this scoping evaluation of the literature since it has been utilized for prior qualitative scoping analyses of literature (15). The literature search was conducted in the PubMed database, as it is a well-established and credible source for research articles.

### 2.2 Search terms

The key search terms used are Visual communication, Data visualization, Interactive visualization techniques, graphical communication, Interactive dashboards, Public Health Data, Medical Data, Clinical Data, Health Data, Health Records, and Big Data. Below is the advanced search detail for the specified key search terms.

((“data visualization”[Title/Abstract] OR “Interactive visualization techniques”[Title/Abstract] OR “visual communication”[Title/Abstract] OR “graphical communication”[Title/Abstract] OR “interactive dashboard”[Title/Abstract]) AND (“Public Health Data”[All Fields] OR “Medical Data”[All Fields] OR “Clinical Data”[All Fields] OR “Health Data”[All Fields] OR “Health Records”[All Fields] OR “Big Data”[All Fields])) AND ((ffrft[Filter]) AND (fha[Filter]) AND (2020:2023[pdat]) AND (English[Filter]))

### 2.3 Study eligibility criteria

The following inclusion and exclusion criteria were used. For an article to be included in the study, it must be (i) peer-reviewed, (ii) have keywords in the title or abstract, (iii) published in English, (iv) user-centered design of visual public health applications/interfaces, (v) involve visualization, visual analytics, infographics, or dashboards of public health data and (vi) published on 2020 and above. A total of 153 articles were included in the initial literature screening that were incorporated in the title and abstract screening. During the title and abstract screening phase, articles were excluded from further review phase if they met any of the following criteria: (i) not peer-reviewed, (ii) published not in English, (iii) technical computer science paper that focused only on algorithms, (iv) full article not available (v) not relevant to the outcomes of interest, (vi) not relevant to visual communication. Also, review articles, commentaries, editorials, posters, systematic and scoping articles were excluded from this review.

In all, 123 (80.4%) articles were excluded after applying the exclusion criteria. A full-text review was conducted on the 30 studies that were left after the exclusion criteria were applied. Upon assessing the whole texts of the remaining 30 research articles, 2 (6.7%) were deemed ineligible due to their failure to address public health visual communication. The review process and selection of articles were done by MAO and NM. The two reviewers screen the 153 articles using the title and abstract. In an instance of disagreement, SR served as a third reviewer to address the differences. This was also done for the full article review. The summary of the entire data extraction form is shown in Figure 1.

### 2.4 Data extraction and reporting

The study used a data extraction form to systematically gather specific details pertaining to descriptive data. Data were collected from each article in other to provide a comprehensive description of the following aspects: the characteristics of the study (author(s), year of publication, title, study type, main theme, and results), visualization tools, form of visualization used, visualization type, and the importance of the visualization used.

## 3 Results

The review focused on the 28 papers that were selected to be included in the study (see Table 1). These papers used different visualization techniques and tools to communicate public health data. The studies represented different aspects of public health including infectious diseases (e.g., HIV, COVID-19) (16–19), non-communicable conditions (20–23), mental health (24, 25), and healthcare system performance (26–28). The analysis involved visualization types, visual displays (graphs and charts used), visualization tools, measures, and public health applications.

**Table 1:** Summary of articles used.

Papesr ID	First author (Year)	Main theme	Visualization tools	Visual displays	Nature and type of visualization	Measure	Public health application
1	Sullivan (2020) (17)	The use of data visualization and dissemination to support HIV prevention and care	Not clearly stated	Heatmap, Bar chart, line graph	Interactive dashboard (AIDSVu)	Accessibility to US AIDS data	Support HIV prevention and care
2	Legenza (2023) (52)	To explore geospatial analysis and data visualization methods among variations in antibiotic susceptibility	ArcGIS	Choropleth map and Heatmap	Static Chart and Graphs	Antibiotic susceptibility and Pattern identification	Decision making, public health intervention
3	Sullivan (2022) (16)	Development and implementation of a data visualization dashboard, America's HIV Epidemic Analysis Dashboard (AHEAD)	Not clearly stated	Line graph, bar chart, and heatmap	Interactive dashboard (AHEAD)	Transparency and accessibility to key data on HIV indicators	Public health communication
4	Brademan (2020) (27)	Provide a user-friendly platform for creating interactive data-hosting websites	Data-driven documents (D3.js)	Volcanos plots, PCA plots, heatmap	Interact web tool (Agronaut)	Interpreting and disseminating large-scale biological data	Rapid recapitulation of results
5	Senathirajah (2020) (53)	Develop methods and visualizations to characterize and quantify display fragmentation and task fragmentation in EHRs	Excel	Sunburst diagram and Time belt	Static chart and graphs	Quantify display fragmentation	Communication of complex HER systems
6	Bell (2020) (54)	Understanding patterns of engagement with the drink less app	R	Heatmap, timeline plots	Static charts and graphs	Identify patterns, measure of associations	Communicate complex patterns from longitudinal data. Public health interventions
7	Sharma (2023) (55)	Development and deployment of an interactive dashboard for visualizing and exploring oral health-related data from national cohort surveys	R	Bar chart	Interactive dashboard	Explore multiple oral health variables	Disseminating health data
8	Vaughn (2021) (19)	To increased symptom awareness, communication, and interpretability of the symptom visualizations	Tableau	Pie chart, bar chart, Heatmap	Static chart and graphs	Increase symptom awareness and interpretability	Public health data management, communication
9	Mollayeva (2022) (56)	Applying data visualization and computational analytics to large healthcare datasets	R	Heatmaps and Bar chart	Static charts and graphs	Explore differences in patterns	Computational analytics and health informatics
10	Chen (2020) (21)	Introduction of TBtools as a comprehensive and user-friendly toolkit for biologists.	Tbtools (JIGplot)	Heatmap, Circos plots, gene structure diagrams	Interactive web-based tool	Visualizing big biological data	Public health intervention
11	Cheng (2023) (24)	Understanding the diagnostic reasoning in medical students through repeated exposures and data visualizations	Not clearly stated	Radial/circular graph	Static charts and graphs	Enhancing diagnostic reasoning with HER	Clinical decision-making, improving medical education
12	Austin (2022) (20)	To explore patterns in whole-person health data	Excel	Bubble charts, parallel coordinates line graphs, box plots, and alluvial flow diagrams.	Static charts and graphs	Explore patterns, measure associations	Public health surveillance
13	Xu (2023) (22)	To develop a comprehensive visualization framework that could effectively communicate and analyze multidimensional oral health survey data	Data-driven documents (D3.js)	Struct view, radar view, cloud view knowledge graph and scatterplot, bar chart	Static charts and graphs	Exploring multidimensional oral health data	Implementation of visual framework for oral health
14	Clarkson (2023) (57)	To examine the importance of context for interpretation and the choice of appropriate visualizations	ArcGIS and tableau	Bar charts, line graph, choropleth maps	Interactive dashboard	Examines the effectiveness of visualizations	Inform best practices, public health communication
15	Balzer (2020) (58)	The introduction of Wiz, a web app that allows users to interactively visualize large datasets	Wiz	Heatmaps, histograms, scatter plots, parallel coordinate plots, bar chart	Interactive web tool	Identifying patterns, trends, and outliers in complex datasets	Public health intervention
16	Grané (2022) (59)	To develop a robust and dynamic method for anal1yzing and visualizing mixed data	R	Box plots, heatmaps, and scatter plots	Interactive web tool	Examining complex relationships and patterns within health-related data	Public health policy measures and decision making
17	Lindroth (2022) (26)	Understanding data visualization and information needs of ICU nurses	Not clearly stated	Bar chart, box plot	Static charts and graphs	Identify information need of ICU nurses	Reduce cognitive load, improve awareness of nurses
18	Bothos (2022) (23)	The development of an integrated platform, SeqCVIBE, for the management, exploration, analysis, and visualization of RNA-Seq data	R	Line graph, bar chart, heatmaps and scatterplots	Interactive web tool	Examine complex genomic data, measure association	Communicate biomedical research, public health interventions
19	Lammons (2023) (60)	Including stakeholders in assessing and reviewing a novel data visualization tool	Not clearly stated	Histogram, bar chart and heatmaps	Interactive dashboard	Reviewing the potential of visualization tool	Evaluation of public health intervention
20	Bowe (2020) (18)	To explore the various data visualization approaches to COVID-19 pandemic	Not clearly stated	Line graphs, bar charts, scatter plots and flowchart and decision trees	Interactive dashboard	Exploring COVID-19 data visualization	Public health surveillance
21	Ericson (2022) (61)	Examine political affiliation perception and interpretation of COVID-19 data visualizations	Not clearly stated	Bar Chart, tree maps and line graph	Static charts and graphs	Examine different visualizations of infectious disease	Public health communication
22	Cocoros (2021) (28)	The development and use of RiskScape as a tool for public health surveillance and data visualization	RiskScape	Heatmaps, bar chart, pie charts, time series plots	Interactive web tool (RiskScape)	To explore patterns, trends, and disparities in various conditions	Public health surveillance, program planning, and evaluation
23	Lin (2023) (25)	To examine the relationships between different mental disorders from the perspective of Traditional Chinese Medicine (TCM)	Not clearly stated	Network visuals, bar charts, and pie charts	Static charts and graphs	Examining connections and distributions of mental disorder	Public health interventions
24	Wanderer (2021) (62)	To evaluate the effectiveness of a data visualization tool that aggregates and presents key anesthesia information	Python	Bar charts, line graph, distribution plot	Static web-based tool (Nimbly)	Improving performance in simulated anesthetic case planning	Clinical decision-making and training
25	Safranek (2022) (63)	To develop a clinical dashboard that enables anesthesiologists to compare their opioid utilization patterns	R and tableau	Stacked bar chart, histograms, box plots, heatmaps	Interactive dashboard	Comparison of opioid utilization patterns	Public health surveillance
26	Wood (2021) (64)	To assess the alignment between real-world clinical practice and the COG protocols for managing SR-ALL in paediatric patients	R	UpSet plots, alluvial graph, time-series plot	Static charts and graphs	Exploring and communicating clinical data. Identifying data gaps	Public health communication
27	Plana-Ripoll (2022) (65)	To Provide a comprehensive atlas of mortality-related estimates associated with a wide range of disorders	Data-driven documents (D3.js)	Bar charts and Line graph	Interactive web page (http://nbepi.com/atlas)	To examine visualization of mortality metrics	Public health surveillance
28	Baxter (2022) (66)	To develop and implement data visualization tool to communicate COVID-19 data	Not clearly stated	Line graph and choropleth maps	Interactive dashboard	Explore complex and evolving COVID-19 data	Public health intervention and decision making

### 3.1 Type and nature of visualization

**Figure 2:**
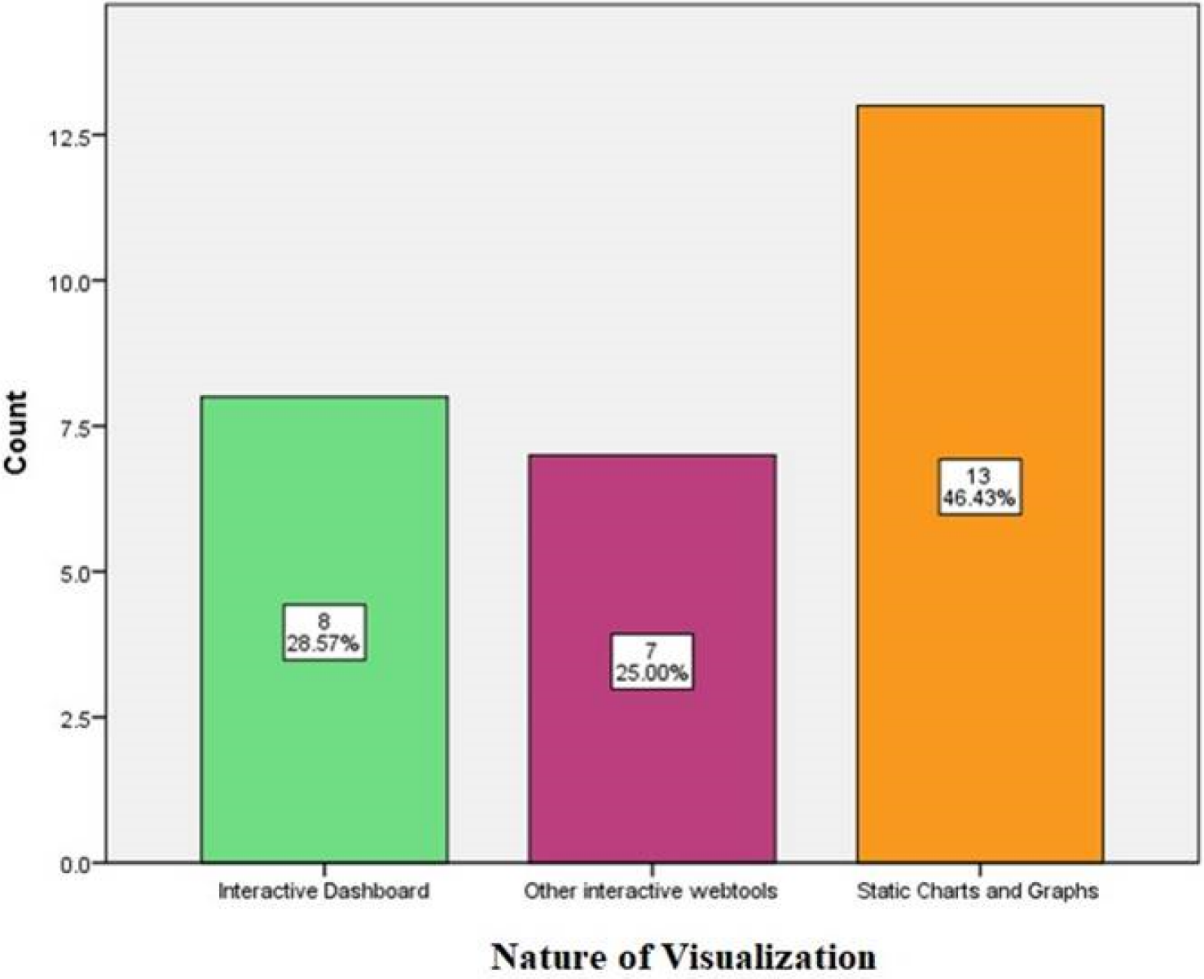
Types and nature of visualization.

Visualizing public health data is very crucial to inform decision-making, as well as to raise awareness and promote health interventions. VC of health data can be static or interactive. The interactive visualizations include interactive dashboards and other interactive web tools and applications. The review revealed that out of the 28 papers that were included, 15 (53.6%) communicated their findings using interactive visualization (1, 3, 4, 7, 10, 14–16, 18–20, 22, 25, 27, 28). The remaining 46.4% communicated their findings visually using static visualizations (2, 5, 6, 8, 9, 11–13, 17, 21, 23, 24, 26). Static visuals like charts and graphs were popular to show specific health data findings or conclusions from the analysis. The evaluation classifies the types of visualizations into three primary categories: interactive web-based applications, interactive dashboards, and static charts and graphs. The review showed that 13 (46.3%) of the studies' visualization falls under charts and graphs (2, 5, 6, 8, 9, 11–13, 15, 17, 21, 23, 26). Interactive dashboards were eight (28.6%) (1, 3, 7, 14, 19, 20, 25, 28). Also, there were seven (25.0%) other web applications (4, 10, 16, 18, 22, 24, 27) (see Figure 2).

The interactive visualizations (interactive dashboards and other web applications) were mostly used in the majority of the studies. According to Figure 2, 53.6% of the studies used interactive visualizations while 46.4% used static charts and graphs. According to Krylov, interactive visualizations provide dynamic data exploration, filtering, and interactivity. However, to effectively communicate certain thoughts or decisions, static representations like charts and graphs are also essential (10).

### 3.2 Visual displays and programming or development tools

There is a broad range of visual displays that was used in the various papers reviewed (see Table 2). These include scatterplots, pie charts, heatmaps, bar charts, line graphs, choropleth maps, box plots, sunburst diagrams, circos plots, network graphics, UpSet plots, and alluvial graphs. The variety of visual presentations emphasizes how crucial it is to use the right visualizations in order to convey many elements of public health data, including distributions, trends, patterns, and correlations. For instance, choropleth maps and heatmaps were used to show spatial patterns of antibiotic susceptibility rates. Alluvial plots and upset plots made it easy to see the alignment of the clinical events with the suggested treatment protocols. Heatmap is used to show patterns in a matrix of data. It requires numerical data in a grid/matrix format which quickly identify high/low values and patterns. Choropleth map displays geographical data with values associated with a geographic region. It visualizes spatial patterns and regional differences. Bar chart comes in different forms and compares quantities across categories. It usually requires categorical data with associated values. It is significantly easy to read and compare discrete quantities. The line graph on the other hand shows trends over time. It requires continuous data, often time series and visualizes trends and changes. The Pie chart shows the composition of a whole and usually requires categorical data to display proportions. Box plot displays distribution of data. It requires numerical data to display median, quartiles, and outliers in a dataset. The scatter plot usually shows the relationship between two variables. It mostly require two sets of numerical data to identify correlations, clusters, and outliers as well.

**Table 2:** Visual displays.

Visuals	Number of studies (%)	Paper ID
Bar chart	18 (64.3)	1, 3, 7, 8, 9, 13, 14, 15, 17, 18, 19, 20, 21, 22, 23, 24, 25, 27
Heatmaps	15 (53.6)	1, 2, 3, 4, 6, 8, 9, 10, 15, 16, 18, 19, 22, 24, 25
Line graph	9 (32.1)	1, 3, 13, 14, 18, 20, 21, 27, 28
Box plot	5 (17.9)	12, 16, 17, 24, 25
Scatter plot	5 (17.9)	13, 15, 16, 18, 20
Histogram	4 (14.3)	15, 19, 24, 25
Pie chart	3 (10.7)	8, 22, 23
Choropleth maps	3 (10.7)	2, 14, 28
Alluvial diagram	2 (7.1)	12, 26
Time series plot	2 (7.1)	22, 26
All others	13 (46.4)	4, 5, 6, 8, 10, 11, 12, 13, 15 20, 23, 21, 26
Circos diagram	1 (3.6)	10
Decision tree	1 (3.6)	20
Sunburst diagrams	1 (3.6)	5
Upset plots	1 (3.6)	26

Alluvial diagram visualizes flow and changes between states. It uses categorical data with changes over time or conditions to depict how categories or states change across multiple dimensions. Circos plot displays complex relationships and genomic data. It utilizes interconnected data often used in genomics and unveils complex relationships in a circular layout. The sunburst diagram shows hierarchical data by displaying multi-level hierarchical data compactly. Lastly, the UpSet plot visualizes set intersections. It utilizes multiple sets and their intersections to analyze complex set relationships more effectively. A detailed review of these visuals are shown in Table 3.

**Table 3:** Detailed review of visuals.

Visuals	Usage	Data	Significance
Heatmap	Showing patterns in a matrix of data	Numerical data in a grid/matrix format	Quickly identify high/low values and patterns
Choropleth map	Displaying geographical data	Values associated with geographic regions	Visualize spatial patterns and regional differences
Bar chart	Comparing quantities across categories	Categorical data with associated values	Easy to read and compare discrete quantities
Line graph	Showing trends over time	Continuous data, often time series	Visualize trends, changes
Pie chart	Showing composition of a whole	Categorical data	Display proportions
Time series plot	Analyzing data points over time	Values associated with specific time points	Identify trends, seasonality
Coxcomb plot (Rose diagram)	Displaying cyclic data or multiple quantitative variables	Categorical and numerical data	Compare multiple variables and show cyclic patterns
Box plot	Showing distribution of data	Numerical data with potential outliers	Display median, quartiles, and outliers in a dataset
Scatter plot	Showing relationship between two or more variables	Two or more sets of numerical data	Identify correlations, clusters, and outliers
Alluvial diagram	Visualizing flow and changes between states	Categorical data with changes over time or conditions	Show how categories or states change across multiple dimensions
Circos plot	Displaying complex relationships and genomic data	Interconnected data, often used in genomics	Visualize complex relationships in a circular layout
Sunburst diagram	Showing hierarchical data	Hierarchical data with parent-child relationships	Display multi-level hierarchical data in a compact form
UpSet plot	Visualizing set intersections	Multiple sets and their intersections	Analyze complex set relationships more effectively

The review underlines how different programming tools are utilized in public health research. The common tools found are R, Tableau, Data-Driven Documents (D3.js), ArcGIS, and Excel (see Table 4). These tools serve as strong and reliable platforms for practitioners and scholars to present public health data in a clear visualization that effectively conveys the intricacies of the information. R is a programming language and software environment provided freely. It was developed for statistical computing and graphics. Originally, R was developed by Ross Ihaka and Robert Gentleman at the University of Auckland, New Zealand, around the mid-90s. R is among the most commonly used tool for data analysis, visualization, statistical modeling in a wide range of sectors, including the finance, healthcare, marketing or the research field (29). The ArcGIS is a geographic information system (GIS) software developed by Esri (Environmental Systems Research Institute). It is a suite of software products used for creating, managing, analyzing, and visualizing geographic data (30).

**Table 4:** Programming or development tools tools.

Visualization tools	Number of studies (%)	Nature of tool	Link	Paper ID
R	7 (25.0)	Public	https://www.r-project.org/	6, 7, 9, 16, 18, 25, 26
Data-driven Documents (D3)	3 (10.7)	Public	https://d3js.org/	4, 13, 27
Tableau	3 (10.7)	Private	https://www.tableau.com/	8, 14, 25
ArcGIS	2 (7.1)	Private	https://www.arcgis.com/index.html	2, 14
Excel	2 (7.1)	Private	https://www.microsoft.com	5, 12
Wiz	1 (3.6)	Private	https://www.wiz.io/	15
RiskScape	1 (3.6)	Public	https://riskscape.org.nz/	22
Tbtools (JIGplot)	1 (3.6)	Public	https://github.com/CJ-Chen/TBtools/releases	10
Python	1 (3.6)	Public	https://www.python.org/	24

Data-Driven Documents (D3.js or simply D3) is an interactive and dynamic data visualization tool for web browsers. It is a JavaScript library for creating dynamic, interactive data visualizations in web browsers. It offers strong capabilities for applying data-driven transformations, generating Scalable Vector Graphics (SVG) components, and connecting data to Document Object Model (DOM) elements (31). Python is a general-purpose, high-level programming language that is extensively utilized for many different kinds of applications, such as automation, scientific computing, web development, data analysis, and machine learning (32).

Tableau is a powerful data visualization and business intelligence software that allows users to create interactive and visually appealing dashboards, reports, and visualizations from various data sources. In terms of data visualization, tableau provides a user-friendly interface for creating a wide range of data visualizations, including bar charts, line graphs, scatter plots, maps, and more (33). Excel is a powerful spreadsheet application developed by Microsoft. It is widely used for data analysis, numerical calculations, data visualization, and a variety of other tasks across various industries. Excel provides a variety of inherent packages and functions for data analysis, such as sorting, filtering, pivot tables, and statistical functions (34). Also, it gives the users the opportunity to develop various types of charts and graphs such as bar charts, scatter plots, line charts, pie charts, and more, to visualize data effectively (35).

### 3.3 Measures

**Figure 3:**
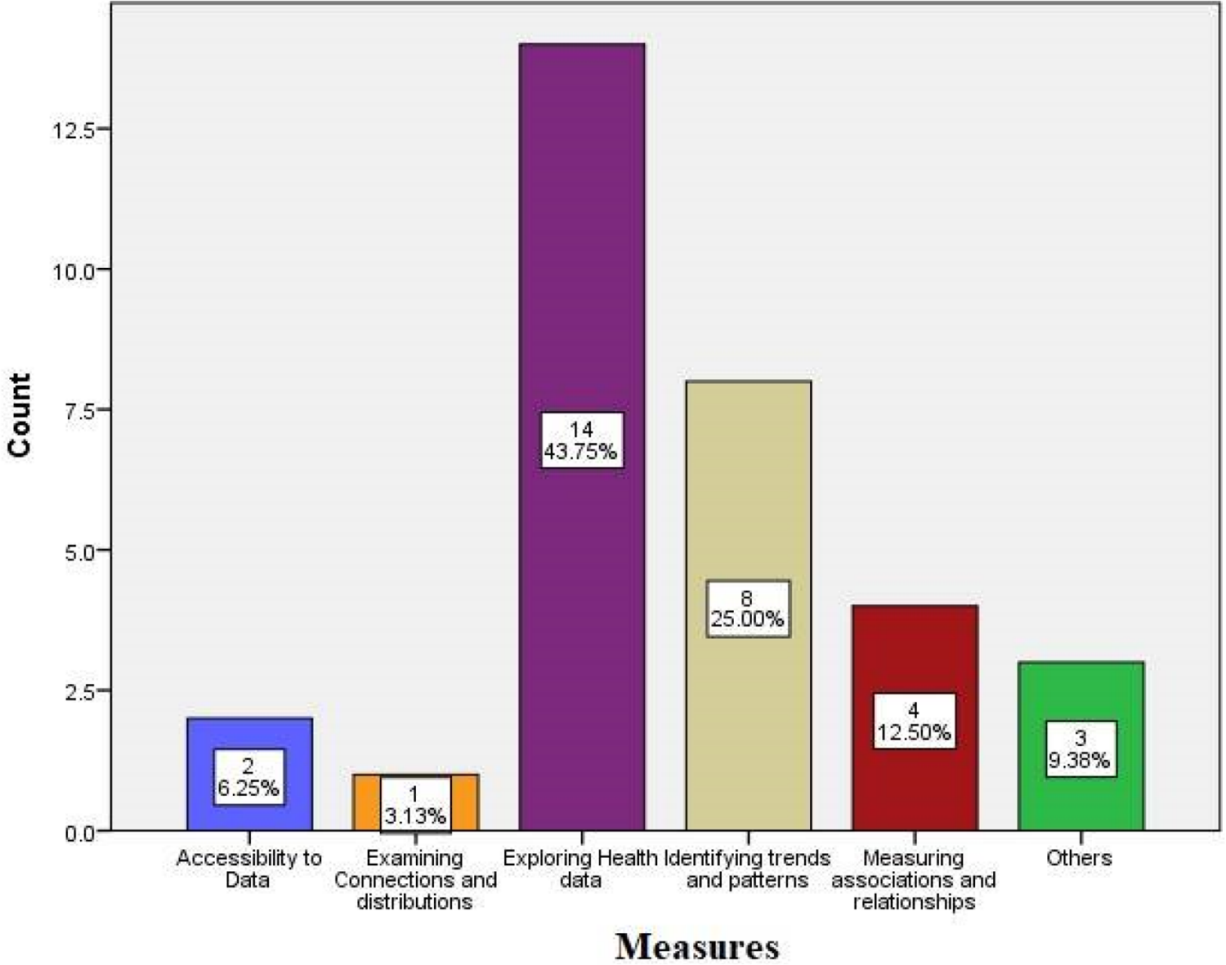
Various measures of data visualization techniques.

The review revealed the various measures and objectives for which the visualizations were employed (Figure 3). These include accessibility to data (1, 3), pattern and trend identification (2, 6, 9, 12, 15, 16, 22, 25), measuring associations and relationships (6, 12, 16, 18), exploring health data (4, 5, 7, 8, 10, 11, 13, 17, 18, 20, 21, 26–28) using different visualizations, reviewing potential of visualization tools (14, 19), examining connections and distributions (23), and identifying data gaps (24). These measures highlight the versatility of data visualization in addressing diverse analytical and communicative needs within the public health domain.

### 3.4 Public health application

Many public health domains, such as HIV prevention and care, antibiotic susceptibility, public health communication, public health interventions, public health surveillance, public health policy measures and decision-making, clinical decision-making, improving medical education, computational analytics and health informatics, biomedical research, program planning and evaluation, and mortality-related estimates, have been the focus of the visualizations found in this review (see Table 5). Effective data visualization is crucial for a wide range of public health research, practice, and communication applications.

**Table 5:** Public health application.

Public health application	Number of studies (%)	Paper ID
Public health communication	9 (32.1)	3, 5, 6, 7, 8, 14, 18, 21, 26
Public health intervention	8 (28.6)	2, 6, 10, 13, 15, 18, 23, 28
Decision making	5 (17.9)	2, 14, 16, 24, 28
Public health surveillance	5 (17.9)	12, 20, 22, 25, 27
Public health policy, planning and evaluation	4 (14.3)	12, 20, 22, 25, 27
Public health education	2 (7.1)	11, 17
Preventive and care	1 (3.6)	1

## 4 Discussion

The visualization of public health data serves as an essential tool to support understanding and conclusion-based decision making for policymakers along with public health professionals as well as other professions. The categories of visualizations used in public health consist of static presentations and interactive interfaces. Static visualizations consisting of printed charts and graphs have been the primary representation tools in public health communication through the years. Recent studies show an increasing interest in interactive visualizations because such interfaces enable users to work with data through dynamic interactions (7) emphasize that static graphics continue to dominate health indicator visualization even though interactive visualizations improve both user capabilities along with their experience. Public health professionals must adjust their data visualization techniques toward emerging technologies that support collection of user requirements (36).

Public health utilizes multiple visual representation tools that include charts, maps and dashboards. These representations serve various purposes, from illustrating complex datasets to facilitating pattern recognition and decision-making. This diversity emphasizes how crucial it is to choose the right visualizations to appropriately depict public health data distributions and trends as well as patterns and correlations (37, 38). The choropleth map is usually used in public health to represent disease distribution data whereas the Community Protection Dashboard developed for COVID-19 allowed stakeholders to monitor real-time information (39). Visual tools for data presentation achieve their goal based on the quality of their design built specifically for the audience's requirements and the data parameters (40, 41).

Programming and development tools play an essential role since they provide practitioners and researchers with platforms to display data effectively and clearly. Various visualization tools mentioned in research literature include R, Tableau, D3.js, ArcGIS, and Excel that help public health data become more understandable to stakeholders (40). Tableau presents an interface designed for easy use by its users to build interactive visualizations for scientific and public health applications (42). The public health agencies benefit from ArcGIS software because it provides effective tools for geographic mapping and population health data tracking (43). Public health informatics education requires the integration of these development tools because they enable students to develop essential expertise for effective data visualization work (40, 44). The choice of a programming tool relies on the visualization job requirements which combine data complexity with the intended audience perspective.

Public health professionals rely on visualizations for three distinct applications in their work: surveillance activities together with policy development and education programs. These tools enable the detection of disease spread and analysis of health patterns while informing policy decisions. The EpiCanvas tool functions as an interactive platform which explores infectious disease data thereby helping public health professionals to monitor and respond to health threats (45). Furthermore, studies on injury surveillance have shown how useful visualizations are for monitoring injury-related incidents, which supports public health initiatives to lower morbidity and mortality (46, 47).

Public health visualizations analyze different types of metrics which include assessment of disease occurrence together with population statistics and health-related social factors. Health disparities monitoring and intervention decision making process depend strongly on these metrics. Through the visual presentation of health determinants Bautista shows how visualized social determinants of health data are useful for creating policy documents that address health disparity issues (38). Public health actions which aim to decrease mortality rates and morbidity benefit from injury surveillance visualizations as demonstrated by (44, 48).

The review demonstrates how public health domains use visualization techniques in public health policy measures, clinical decision-making and computational analytics and mortality-related estimate (49, 50). Visualizations contribute to understanding data better while they aid policymakers to establish evidence-based procedures and policies. The COVID-19 pandemic monitoring process depends heavily on interactive dashboards which track live data enabling public health authorities and communities to take better actions (50, 51). Public health practice depends heavily on data visualization. Given the increasing complexity of public health data and the challenges associated with them, it is crucial to have well-designed visualizations that effectively communicate important information.

## 5 Limitations

The comprehensive findings from this literature review should be considered alongside its limitations. One limitation is that only the PubMed database was searched, so any relevant studies published outside of PubMed may have been missed. Again, there are other gray literature that would have been a great benefit that we did not consider because of our inclusion criteria. Another potential limitation relates to the specific keywords used when searching for articles. It is possible that using a different set of keywords could have surfaced additional relevant studies that were overlooked. Furthermore, this review only included studies written in English, excluding any non-English papers that could have provided further insights. To try to reduce bias, the references of all included studies were manually screened.

## 6 Conclusion

The field of public health data visualization continues to develop by combining static and interactive visualization techniques, which are crucial for conveying intricate health information. As static visualizations continue dominant, interactive tools are gradually becoming relevant due to their ability to enhance user engagement and data exploration. The selection of suitable visualization method either through charts, maps or dashboards needs to match the needs of the target audience and match data complexity while meeting desired outcomes.

Public health professionals rely on programming and development tools which include R, Tableau, D3.js, ArcGIS and Excel to produce effective data communications for diverse stakeholders. These developing tools enable critical public health functions including disease surveillance, policy development, and education programs. The COVID-19 pandemic has particularly highlighted the value of real-time interactive dashboards for monitoring and response.

As public health challenges grow increasingly complex, the importance of well-designed data visualizations becomes more pronounced. Future directions should focus on integrating emerging technologies, improving user requirement collection, and enhancing visualization techniques to better address health disparities and support evidence-based decision-making. Public health professionals must continue adapting their visualization approaches to meet evolving needs and leverage new tools that can effectively communicate vital health information to diverse audiences.

## Data Availability

The original contributions presented in the study are included in the article/Supplementary Material, further inquiries can be directed to the corresponding author.
